# Moving beyond mean heart dose: The importance of cardiac substructures in radiation therapy toxicity

**DOI:** 10.1111/1754-9485.13737

**Published:** 2024-09-03

**Authors:** Sarah Bowen Jones, Tom Marchant, Chris Saunderson, Alan McWilliam, Kathryn Banfill

**Affiliations:** ^1^ Radiotherapy Related Research The Christie NHS Foundation Trust Manchester UK; ^2^ Division of Cancer Sciences University of Manchester Manchester UK; ^3^ Department of Cardiology, Leeds General Infirmary Leeds Teaching Hospitals NHS Trust Leeds UK

**Keywords:** cardiotoxicity, radiation oncology

## Abstract

Normal tissue tolerance dose limits to the heart have been established to reduce the risk of radiation‐induced cardiac disease (RICD). Dose constraints have been developed based on either the mean dose delivered to the whole heart (MHD) or the dose delivered to a specific volume, for example, volume of heart receiving equal to or greater than 30 Gy (V30). There is increasing evidence that the impact of thoracic radiation on cardiac morbidity and mortality has been underestimated. Consequently, there is a need to reduce the dose delivered to the heart in radical radiotherapy treatment planning. The pathophysiology of RICD may relate to dose to specific cardiac substructures (CS) rather than the traditionally observed MHD for common toxicities. The MHD or V30 Gy threshold dose rarely represents the true dose delivered to individual CS. Studies have shown the dose to specific areas may be more strongly correlated with overall survival (OS). With advances in modern radiotherapy techniques, it is vital that we develop robust, evidence‐based dose limits for CS, to fully understand and reduce the risk of RICD, particularly in high‐risk populations with cardiac risk factors. The following review will summarise the existing evidence of dose limits to CS, explain how dose limits may vary according to different disease sites or radiation techniques and propose how radiotherapy plans can be optimised to reduce the dose to these CS in clinical practice.

## Introduction

Dose constraints exist in radiotherapy to protect normal tissue from the harmful effects of radiation. The Quantitative Analysis of Normal Tissue Effects in the Clinic (QUANTEC) guidelines include normal tissue tolerance dose limits using dose‐to‐volume response models based on outcome data.^1^ There are limitations of applying the QUANTEC guidelines to develop cardiac dose constraints, as the guidelines were developed based on data from patients receiving radiotherapy for oesophageal cancer or to the mediastinum for lymphoma. These treatments involved lower doses of radiation and utilised older, less conformal radiotherapy techniques compared with modern radiotherapy practices. In addition, they did not account for the effect of systemic anti‐cancer agents that can induce cardiotoxic effects.

Chemotherapy agents, targeted therapies, monoclonal antibodies and immunotherapy increase the risk of radiation‐induced cardiac disease (RICD) when administered prior to or in combination with radiotherapy.^2^, ^3^ MHD is an imprecise surrogate marker for dose to cardiac substructures (CS) in patients receiving thoracic radiotherapy. The importance of dose to individual CS is increasingly recognised and to fully understand and minimise RICD for patients, there is a need to determine which CS are the most critical for limiting resulting cardiac morbidity and mortality.

This focused review will summarise the current array of evidence relating RICD to CS, including suggested dose parameters. Evidence is summarised from studies including patients with breast, lung and oesophageal cancer.

## Limitations of whole heart dose parameters

Heart dose limits in thoracic radiotherapy have traditionally been based on dose received by the whole heart contour. MHD is the most referenced dosimetric parameter in national radiotherapy guidelines.^4^ Several trials have demonstrated a link between higher heart radiotherapy dose and risk of cardiac morbidity and mortality in breast, lung, oesophageal and lymphoma cancer survivors.^5^ Major adverse coronary events (MACE) recorded in these studies are comprised of myocardial infarction, hospitalisation for heart failure, coronary revascularisation or cardiac death. Seminal papers in patients treated for breast cancer and Hodgkin's lymphoma showed a linear dose–response relationship between radiation dose to the heart and the risk of coronary heart disease or MACE.^6^, ^7^ With each additional 1 Gy delivered to MHD, there was a 7.4% relative increased risk of MACE or coronary heart disease risk. The first prospective trial in patients with lung cancer that demonstrated a link between heart dose and cardiac toxicity was the RTOG‐0617 trial.^8^ Patients receiving 74 Gy in the dose‐escalated arm had worse OS compared with the 60 Gy control arm. Further analysis showed that the most significant factor associated with worse OS on multivariable analysis after controlling for known prognostic factors, was the volume of heart receiving over 40 Gy (HR 1.012, *P* =< 0.001).^9^

It is not possible to use whole heart dose parameters to differentiate variations in sensitivity of different CS, as the MHD does not facilitate differentiation between uniform irradiation and highly heterogenous distribution of dose. Dose‐volume parameters such as V30 Gy can be used to characterise the subvolumes of the heart receiving high dose more accurately, although spatial information is not captured, making it difficult to link the precise area of the heart that is irradiated with the resulting toxicity. For example, from MHD reporting it cannot be determined what dose was delivered to the coronary arteries in those with a resulting ischaemic event. The dose delivered to the coronary arteries may also be underestimated by current MHD metrics. In the BACCARAT study of breast radiotherapy, despite a MHD <3 Gy, 56% of patients received a dose to the left anterior descending artery (LAD) of more than 40 Gy.^10^

## Mechanisms of toxicity

The pathophysiology behind RICD is complex and remains to be fully explained but is thought to encompass several mechanisms. Cardiac toxicity varies according to the CS and its response to radiotherapy.^11^ Radiotherapy causes direct and indirect DNA damage leading to apoptosis of cardiac myocytes, mitochondrial dysfunction due to oxidative stress, cytokine release, endothelial injury and microvascular dysfunction. Furthermore, fibrosis and calcification of cardiac valves results in valvular heart disease. Fibrosis of the myocardium and reduced cardiac contractility is associated with heart failure, whereas fibrosis of conduction tissue leads to arrhythmias. Inflammation in the endothelial layer of coronary arteries also contributes to accelerated atherosclerosis within coronary arteries.

### Pericardial disease

Pericardial complications such as pericardial effusion and pericarditis are observed in response to radiation‐induced inflammation, microvascular damage and impaired lymphatic drainage from the pericardium.^12^ Studies evaluating dosimetric risk factors for symptomatic pericardial disease have found the dose to the whole pericardium (V30, V55, V80 or MHD) to be of particular significance. As the pericardium is thought to function as a parallel organ, pericardial disease has been significantly associated with dose to whole heart dosimetric parameters.^13^, ^14^, ^15^ The QUANTEC guideline dose constraint recommendation for the pericardium is V30 <46%.^1^

### The atria and conduction system

The right atrium receives deoxygenated blood from the body via the superior and inferior vena cava. The left atrium receives oxygenated blood via the pulmonary veins from the lungs and directs it towards the left ventricle. The atrial myocardium is thin compared to ventricles due to a lower force required to contract and expel blood the relatively short distance into the ventricles. The atria contain the sino‐atrial and atrio‐ventricular nodes and thus have an important role in cardiac conduction. The sino‐atrial node (SAN) is a 10–20 mm spindle‐shaped structure located on the upper posterolateral wall of the right atrium at the junction with the superior vena cava.^16^ Located within the SAN are nodal myocytes that automatically depolarise to generate electrical impulses and thus form the natural pacemaker of the heart. Once the action potential is generated within the SAN it propagates through cardiomyocytes to the atrio‐ventricular node (AVN), which is in the posteroinferior region of the interatrial septum near the coronary sinus, via intra‐atrial muscle bundles. Simultaneous rapid interatrial conduction primarily via muscular connections to the left atrium (LA) enables synchronous biatrial contraction.^17^

The pathophysiology of atrial fibrillation (AF) is complex and beyond the scope of this review but atrial fibrosis as well as triggered activity from the proximal pulmonary veins are implicated in arrhythmogenesis and initiation of atrial fibrillation.^18^ The development of AF after chemoradiotherapy (CRT) has been reported in patients with non‐small cell lung cancer (NSCLC) and small cell lung cancer (SCLC).^19^ A retrospective study of 239 patients with limited stage SCLC and 321 with locally advanced NSCLC found the 3‐year cumulative incidence of AF was higher in those in the SCLC group who received a dose to the SAN >53.5 Gy compared with those who received <53 Gy (25% vs 2%, *P* =< 0.001). The SAN Dmax was predictive of 3‐year cumulative incidence of AF in both groups. The SAN Dmax >20 Gy was predictive in NSCLC, whereas in SCLC there was a higher predictive dose, Dmax >53.5 Gy. This suggests a possible radiosensitising effect of paclitaxel chemotherapy in the NSCLC group, leading to a lower radiation dose threshold for arrhythmic toxicity. On multivariable analysis, the 3‐year OS was also significantly lower in groups who received higher doses to the SAN (SCLC HR 2.68, 95% CI 1.53–4.71, *P* =< 0.001, NSCLC HR 1.97, 95% CI 1.45–2.68, *P* =< 0.001). This association was not seen with other cardiac events. Nevertheless, the number of AF events in this study was low with only three events in the SCLC cohort and eight in the NSCLC cohort.

New AF has been observed in patients with oesophageal cancer after neo‐adjuvant CRT. A retrospective study including 238 patients, found the incidence of AF at 1 year was 19.5% and median time to development of AF was 4.1 months.^20^ This study conducted detailed recording of cardiac events. The observed incidence of AF in this study was higher than other studies comparing AF incidence after oesophagectomy without radiotherapy, especially using minimally invasive techniques. Increased dose to the LA was associated with reduced OS on multivariable analysis. LA V20 >84%, was associated with worse OS compared with LA V20 <84% (HR 1.63, 95% CI 1.17–2.28, *P* = 0.04). For each 10 Gy increase in dose to LA, there was a 30% increase in AF risk. The mean LA dose was 35.6 Gy (31.1–43.4 Gy), and right atrium (RA) mean dose was 26.6 Gy (range 20.2–34.5 Gy). The risk of MACE was 25.7% at 1 year, and the rate of cardiac death was 3.8% in this cohort.^21^

The dose to the conduction system has also been evaluated in patients undergoing SBRT for central thoracic tumours by contouring the SAN and AVN in 93 patients, allowing Dmax and Dmean for each structure to be calculated. The median dose to SAN Dmax was 9.47 Gy, and median Dmean 5.75 Gy. On multivariate analysis, the SAN values were significantly associated with worse OS rather than AVN (Dmax HR 2.03, *P* = 0.026 and Dmean HR 2.22, *P* = 0.011, respectively).^22^ There are no studies that consider the dose to the AVN as having a significant impact on cardiotoxicity or arrhythmias. Due to the more central position of the AVN within the heart, it has been shown to receive a lower dose during radiotherapy for lung cancer.^19^, ^22^

The close proximity of the right and left atria may lead to a high correlation between doses to the different parts of the conduction system within the atria. A retrospective analysis of ECGs taken within a radiotherapy dose escalation study in patients undergoing concurrent chemoradiotherapy for NSCLC found that those who received high LA wall radiation dose (>63 Gy to >2.2%) had significantly higher death rate (HR 2.55, CI 1.10–5.95, *P* = 0.03). The 2‐year survival was higher in patients whose LA received <63 Gy (81 vs 58%).^23^ Another study evaluating non‐cancer death following stereotactic ablative radiotherapy (SABR) for early‐stage NSCLC, also found that the dose to the LA (median dose 6.5 Gy, HR 1.005, *P* = 0.035) was particularly significant.^24^ The most important CS within the atria are the different parts of the conduction system, therefore, the atrial dose acts as a surrogate for dose to SAN or AVN. The conduction system is not visible on CT and must therefore be outlined using surrogate anatomical landmarks.

### Coronary arteries

The coronary arteries originate from the ascending aorta. Thoracic radiotherapy causes oxidative stress and chronic inflammation in coronary arteries.^11^ This leads to accelerated atherosclerosis, coronary artery plaque rupture or acute thrombotic occlusion, which results in cardiac myocyte necrosis in the territory supplied by that artery. Consequently, coronary artery damage can impact on other CS. The right coronary artery (RCA) traverses the surface of the right atrium to supply blood to the right atrium, left and right ventricle, SAN and AVN.^25^ The left main coronary (LMC) artery traverses the LA and divides into the left circumflex artery and left anterior descending (LAD) artery to supply the left atrium and ventricle.

Due to the proximity of the LAD to a breast radiotherapy tangent pair, most studies on coronary artery dosimetry have been focussed on patients with breast cancer. A dosimetric analysis of patients with breast cancer found the maximum and mean dose to the LAD artery was associated with major cardiac events on multivariable regression analysis.^26^ Dose to the LAD was also found to correlate with a significantly higher rate of arrhythmias in left, compared with right sided breast cancers (14% vs 1% *P* = 0.001). The equivalent dose to the LV, RV and LAD were significantly associated with a resultant decrease in LVEF >10% (*P* = 0.37, *P* = 0.023 and *P* = 0.049, respectively). These results suggest that coronary artery damage can lead to myocardial hypoperfusion and impact cardiac contractility.^27^

The risk of coronary artery stenosis following breast radiotherapy has been evaluated in 649 women undergoing percutaneous coronary intervention (PCI). In those who had received left‐sided RT, there was a significantly higher risk of requiring PCI to the LAD (HR 1.44, 95% CI 1.12–1.77, *P* =< 0.001). The risk was highest in the distal LAD in women with more advanced nodal disease involvement who would have been treated with an extended radiation field (HR 2.43, 95% CI 1.33–4.41).^28^

There is also evidence of coronary artery damage following radiotherapy for patients with lung and oesophageal cancer. In further evaluation of the RTOG‐0617 trial data set, 79% of patients received LAD V15 Gy >10%, and when compared to those receiving <10% there was significantly higher all‐cause mortality (HR 1.43, 95% CI 1.02–1.99, *P* = 0.037). Conversely, MHD did not have the same mortality association. There was a statistically significant difference in median OS (20.2 vs 25.1 months) if treated LAD V15 Gy >10%, compared with <10%.^29^

A retrospective analysis of 700 patients by Atkins *et al*. treated for NSCLC found that when adjustment was made for baseline coronary heart disease and prognostic factors, V15 Gy to LAD >10% correlated with higher risk of MACE events (adjusted HR 13.09, 95% CI 1.23–157.21, *P* = 0.03) and all‐cause mortality (adjusted HR 1.58, CI 1.09–2.29, *P* = 0.02). This was particularly evident in patients without underlying coronary heart disease at baseline. Conversely, the LV V15 Gy >10% most strongly correlated increased risk of MACE in patients with underlying CHD.^30^

In two studies involving patients with oesophageal cancer undergoing concurrent CRT, the dose to the coronary arteries was demonstrated as being more important than either MHD or heart V30 Gy. Wang *et al*. reported 14 major cardiac events in a study which involved 355 patients. These events were found to be significantly associated with the volume of LAD receiving >30 Gy (*P* = 0.048).^31^ The relative rate of death increased with left main coronary artery mean dose (*P* = 0.002). A significantly increased risk of major cardiac events was also observed in those with pre‐existing hyperlipidaemia, which is a recognised risk factor for cardiovascular disease (*P* = 0.007).^32^ Cai *et al*. studied 716 patients, 68 experienced a grade three (G3) cardiac event. On multivariable analysis, dose to LV, LAD and left circumflex (LCX) were associated with G3 toxicity. They suggested a predictive model which included LAD and LCX dose and found that this was significantly predictive of cardiac events, specifically ACS and CHF, whereas the whole heart dose model was not predictive.^33^ However, the paper does not suggest dose limits to these CS.

The effect of baseline cardiac comorbidity was reviewed in a retrospective dosimetry review of 140 patients with inoperable NSCLC treated with CRT by Yegya‐Raman *et al*.^34^ Patients were categorised according to presence of coronary artery disease (CAD). Cardiac events occurred from 15.3 months onwards, and the 4‐year cumulative incidence of MACE was 23.4% in those without baseline CAD versus 51.9% in those with CAD. The LAD and ventricle doses were associated with acute coronary syndrome and heart failure on multivariable analysis after pairing for baseline CAD (adjusted HR 1.042, 95% CI 1.018–1.066, *P* = 0.0005). There was no evidence to support the dose to whole heart or atria being associated with arrhythmias.

History of CAD can be identified from patient notes however, there will be patients who have undiagnosed CAD. The degree of calcium within the coronary arteries identified on baseline radiotherapy planning CT scan (CAC score), has been identified as a predictor of cardiovascular events in patients with breast cancer undergoing adjuvant radiotherapy. This has also been shown in patients with lung cancer, where volume of left coronary artery receiving 15 Gy and CAC score were independently associated with cardiac events.^35^ A reduction in the mean right coronary artery dose significantly reduced the risk of accelerated CAC burden in this group of patients.^36^

### Ventricles

The ventricles are responsible for pumping blood from the heart around the body. The contraction of ventricular myocytes is co‐ordinated by electrical impulses from the AVN, transmitted through the Bundle of His and specialised Purkinje fibres in the sub‐endocardium to the ventricular walls. This generates powerful contraction of the ventricles and forces blood out through the outflow tracts. The right ventricular outflow tract directs blood through the pulmonary arteries to the lungs, and the left ventricular outflow tract directs blood via the aorta to the rest of the body.

A recent multimodality imaging study used cardiac magnetic resonance (CMR) imaging and echocardiography to analyse changes to the myocardium in patients with left‐sided, early‐stage breast cancer treated with radiotherapy. Imaging was performed at baseline, after RT, at 3‐ and 6‐year follow‐up appointments. The highest radiation doses were observed in the apical and anterior area of the left ventricle (Mean dose 32 Gy). A higher left ventricle (LV) radiation dose was associated with myocardial fibrosis in the apical and septal regions, on both imaging modalities (OR 1.26, 95% CI 1.00–1.59, *P* = 0.0047).^37^ In a dosimetry study of breast radiotherapy, the dose to the anterior LV wall was considerably higher than MHD (median dose 9 Gy). The median Dmax to the apical region of the ventricle was 15 Gy (range 3–24 Gy). The LAD also received a high mean dose 18 Gy (range 4–30 Gy) and max dose 41 Gy (range 18–44 Gy). It is important to understand the true dose distribution in breast radiotherapy, in patients who are traditionally thought to receive lower cardiac doses.^38^

Perfusion studies have shown ischaemic defects in the LV of patients following left‐sided breast radiotherapy, at a rate of 40% at 2 years, and which were significantly higher if over 5% of the volume of the LV was treated.^39^ A multivariable analysis by Lai *et al*. of patients receiving adjuvant breast radiotherapy found that the best predictor for ischaemic cardiac events was LV V25 >4%. (HR 2.12, 95% CI 1.11–4.03, *P* = 0.023) This dose constraint was exceeded in 45% of the patients with left sided breast cancer in the study, despite heart sparing techniques such as deep inspiratory breath hold (DIBH) being available. Patients who were above this threshold were at twice the risk of developing a major ischaemic event, compared with those who did not (HR 2.22, 95% CI 1.21–4.05, *P* = 0.010). The LV model was a better predictor than MHD for major ischaemic events. In this analysis, dose–volume parameters for coronary vessels specifically LAD were not significant.^40^

In a retrospective study involving patients with stage III NSCLC, the volume of LV receiving >60 Gy >0 was significantly associated with acute coronary syndrome (ACS) (SHR = 9.49, 95% CI = 1.28–70.53, *P* = 0.028) in those with pre‐existing cardiovascular risk factors. Unlike the previous study mentioned by Atkins *et al*., and in keeping with the research by Yegya‐Raman *et al*., this association was higher in those with underlying cardiovascular risk factors.^41^ There is conflicting evidence in the literature regarding whether baseline cardiac comorbidity warrants a greater degree of cardiac sparing and future studies may be needed to explore this in order to develop recommended dose constraints for high‐risk patients.

The right ventricle has also been shown to be of importance in a study of NSCLC patients treated with CRT. The mean right ventricle dose >5.5 Gy was associated with a higher risk of death without progression (HR 1.08 1.04–1.11, *P* = <0.001).^42^

Valvular heart disease has been observed after radiation treatment. In patients receiving radiotherapy for thoracic cancers (*n* = 243) (Hodgkin's lymphoma, breast, lung and oesophageal malignancy), compared with matched controls, significantly more patients in the RT group underwent aortic valve replacement, with higher mortality in the RT group at mean follow up 6.6 years (40% vs 11% *P* =< 0.001).^43^ Most evidence regarding valvular heart disease has been obtained from studies of Hodgkin's lymphoma or paediatric cancer survivors, and the prevalence of valvular heart disease, which has previously been thought to have a long latency period, increases with time.^44^, ^45^, ^46^

### Composite structure

Most studies of CS rely on contouring of the relevant structures, which can be inconsistent. A large study by McWilliam *et al*. used an image‐based data mining (IBDM) technique to define an area of the heart most sensitive to radiation. This technique did not rely on contouring of CS, but instead comprised a data‐led approach which associated radiotherapy dose against survival in 1100 patients. An area at the base of the heart was identified as the area whereby excess radiation dose correlated with worse OS at 12 months (HR 1.25, 95% CI 1.01–1.56, *P* = 0.04). This area corresponded to a composite structure encompassing the proximal aorta, origin and proximal coronary arteries, SAN and right atrium. In this study, other whole heart dose parameters MHD, V5 and V30 did not predict survival.^47^ Two further studies using IBDM on data from randomised clinical trials have further validated this association. The radiotherapy plans of patients in the RTOG‐0617 trial,^48^ and the PET‐PLAN trial^49^ were analysed, and in both trials the dose to this area at the base of the heart region was found to be significantly associated with OS.

In another study by McWilliam *et al*., 14 different CS were delineated by registering radiotherapy planning CT images onto five template anatomies, to calculate mean and max dose to each CS and to minimise the effect of outliers on dose distribution. Variable reduction techniques were used to determine the area of the heart that was most sensitive to radiation. The maximum dose to the RA, right coronary artery and ascending aorta were selected as the CS most critical for OS on multivariable analysis (HR 1.01, *P* = 0.03) after accounting for tumour size, performance status and nodal stage. Patients who received >19.5 Gy to this area had an OS of 12 months compared with 21 months in those who received <19.5 Gy to this area.^50^ These results were validated in the NI‐HEART study in patients receiving radiotherapy for NSCLC (*n* = 478), whereby dose to the base of the heart was associated with cardiac events and adverse survival after adjusting for clinical characteristics and baseline cardiac risk factors on multivariate analysis (HR 1.75, CI 1.03–2.97, *P* = 0.04, and HR 1.40 95% CI 1.14–1.75, *P* = 0.0017, respectively).^51^

## What is the evidence for the dose constraints to these cardiac substructures?

Existing evidence has not reached a consensus for optimum dose constraints to each CS. Table 1 below summarises the existing recommended dose constraints to CS.

**Table 1:** Summary of evidence for dose constraints to cardiac substructures

Reference	Disease site	No of patients	Dose constraint	Outcome	Dose and fractionation	SACT
Atria (Including base of heart, pulmonary artery, pulmonary veins, superior vena cava, SA node)						
McWilliam 2020^50^	Lung	971	Base of the heart Max dose <19.5 Gy	OS	55 Gy/20#	Induction chemotherapy in 218 patients
Stam 2017^24^	Lung	803	Max dose to LA and D90% SVC	Non‐cancer death	SBRT 54 Gy/3# or 48 Gy/4#	None
Ma 2017^52^	Lung	141	Pulmonary artery V45 Gy (68–70%)	OS	60–74 Gy/30–36#	CRT or RT alone permitted
Lovoli 2024^22^	Lung	93	Cutoff values SAN Dmax 13 Gy, SAN Dmean 8 Gy	OS	SBRT >50 Gy/5#	None
Kim 2022^19^	Lung	560	SCLC SAN Dmax >53.5 Gy, NSCLC SAN Dmax >20 Gy	3 year incidence of AF	60–63 Gy/1.8–2 Gy per #	Concurrent chemotherapy
Walls 2024^53^	Lung	420	Left PV V55 < 2% Right PV V10 < 54%	AF	55 Gy/20#	CRT or RT alone permitted
Miller 2023^21^	Oesophagus	238	LA V20 >84%	OS	1.8‐2Gy per #	Concurrent chemotherapy
Ventricles						
Chan 2020^54^	Lung	112	RV D4% <11 Gy optimal (13.3 Gy mandatory)	OS	SBRT 54 Gy/3# or 50 Gy/4–5# (central tumours)	None
Yega‐Raman 2023^42^	Lung	187	RV mean dose ≥5.5 Gy	DWP	60–74 Gy/30–37#	Concurrent chemotherapy
Jang 2020^41^	Lung	258	LV D 0.1 cm^3^ < 60 Gy (In high baseline cardiovascular risk)	ACS	50–74 Gy/20–36#	Concurrent chemotherapy
German Society of Radiation Oncology ‐ DEGRO^55^	Breast	N/A	LV Dmean <3 Gy V5 <17% V23 <5%	Not defined	50 Gy/25#	None
Van den Bogaard^56^	Breast	910	LV V5 (no dose cut off)	ACE	50.4 Gy/28# +/− boost 14–16.8 Gy	None
Jacob 2019^10^	Breast	104	LV V20 <15%	LV dysfunction	50 Gy/25, or 47 Gy /20#, +/− boost 9–15 Gy	None
Lai 2023^40^	Breast	2,158	Recommended LV V25 <4%	Major ischaemic event	45–50.5 Gy +/− boost 10–16 Gy /20–28# +/− boost 10–16 Gy /5–8#	None
Coronary arteries						
Atkins 2021^30^	Lung	700	LAD V15 <1% (With underlying CVD) LAD V15 <10% (Without underlying CVD) LCX V15 Gy <14% LV V15 Gy <1% Mean total coronary artery dose <7 Gy	MACE events and all‐cause 2‐year mortality	50–66 Gy/25–33#	Induction or concurrent chemotherapy
No 2023^35^	Lung	233	Total LAD receiving >15 Gy	Cardiac events ≥grade 3	Definitive radiotherapy	Permitted
Zureick 2022^26^	Breast	375	LAD mean < 3 Gy	Cardiac events	50–50.4 Gy/25–28# or 42.5 Gy /16# +/− photon or electron boost 10–12.5 Gy/4–5#	None
Thomsen 2021^57^	Breast	1,182	LAD Dmax <20 Gy (50 Gy regimen) Dmax <17 Gy (40 Gy regimen)	Late cardiac morbidity	50 Gy/25# 40 Gy/15#	None
German Society of Radiation Oncology – DEGRO^55^	Breast	N/A	LAD Dmean <10 gy V30 LAD <2% V40 LAD <1%	Not defined	50 Gy/25#	None
Cai 2022^33^	Oesophagus	716	LAD V30 LCX V45	G3 ACS and CHF events	50–50.4 Gy/28#, boost to 66 Gy if dose to OAR dose constraints met	Sequential or concurrent CRT

ACE, acute coronary event; ACS, acute coronary syndrome; AF, atrial fibrillation; CHF, chronic heart failure; CRT, chemoradiotherapy; DWP, death without progression; Gy, grey; IMRT, intensity‐modulated radiotherapy; LAD, left anterior descending; LA, left atrium; LCX, left circumflex; LV, left ventricle; MACE, major adverse coronary events; OAR, organs at risk; OS, overall survival; PV, pulmonary vein; RT, radiotherapy; RV, right ventricle; SBRT, stereotactic ablative radiotherapy; SVC, superior vena cava; #, number of fractions.

## Which structures are important for each disease site?

**Fig. 1:**
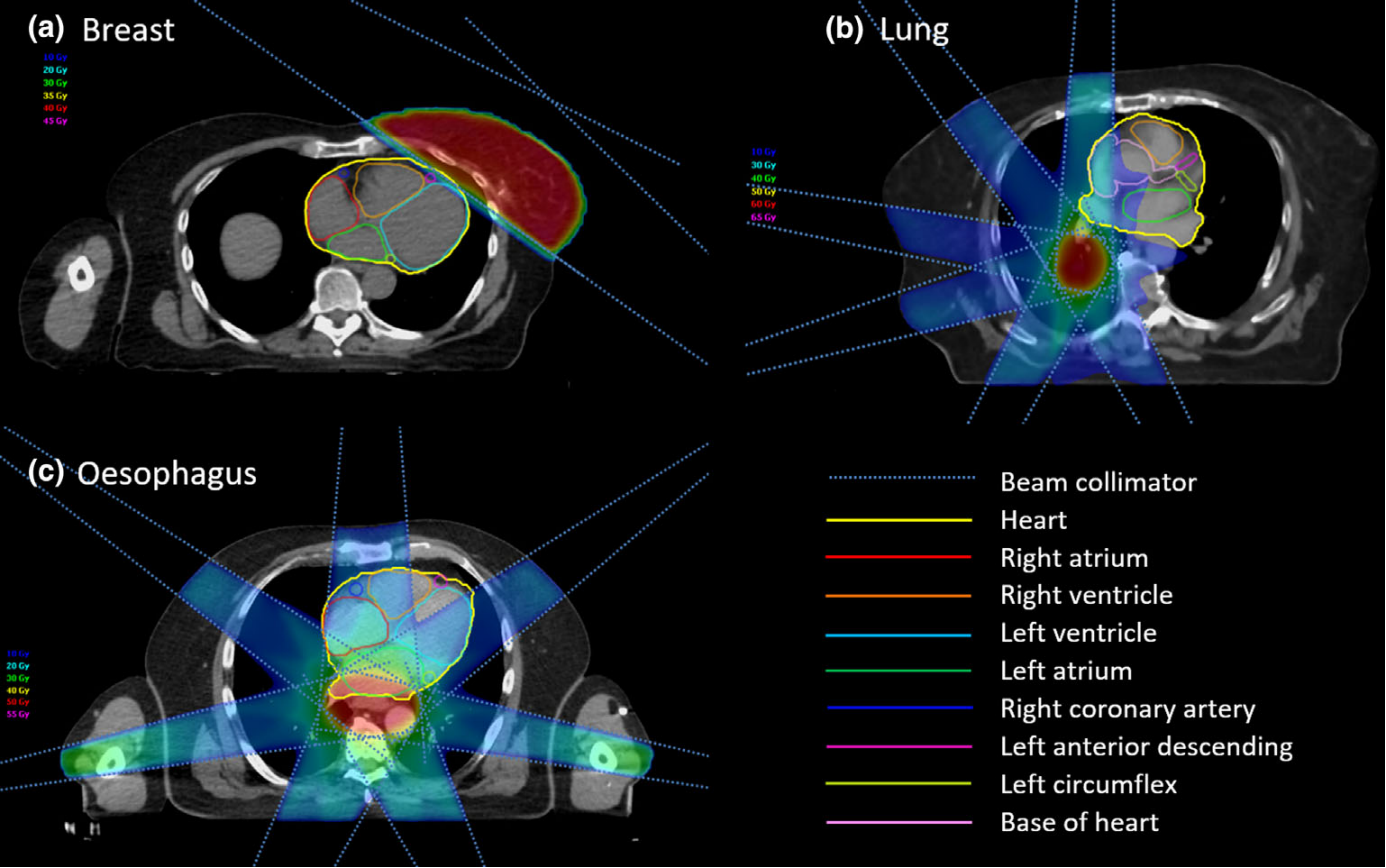
IMRT treatment plans for representative patients with (a) breast, (b) lung and (c) oesophagus cancer. Cardiac substructures are outlined as per key, beam arrangement and dose distribution illustrated for each disease site.

Figure 1 presents the CS included in the radiation field which vary according to disease site. The varying dose deposition is due to different radiotherapy field arrangements, nearby organs at risk, disease location and patient‐related factors.

### Breast

A tangential field arrangement is used in patients with breast cancer using conventional or field‐in‐field intensity‐modulated radiation therapy (IMRT). This beam arrangement ensures coverage of the breast or chest wall and includes a small volume of lung and anterior portion of the heart. Consequently, the LAD coronary artery and ventricular apex may receive a high dose of radiation, especially in left breast irradiation. Where regional nodal irradiation is required, the field is extended to cover the axilla and/or superior clavicular fossa. Additional beams are added in a conformal manner if the internal mammary chain of lymph nodes is included. Extending the fields and including the nodal areas increases the dose to CS as the field size and depth increases. Dose to CS also depends on patient factors such as breast size, laterality, chest wall separation, heart size (both anatomical and the extent of cardiac contour) and radiation factors such as whole breast versus partial breast treatment, hypofractionation and boost dose schedules. Techniques to reduce the heart dose such as DIBH are widely used to exclude as much of the heart from the radiotherapy field as possible. DIBH has been shown to reduce the maximum dose to the LAD (LADmax) by 19.8%.^58^

### Lung

There is heterogeneity of dose distribution across the thorax and across CS in patients with lung cancer depending on the location of primary and nodal disease. IMRT techniques use inverse planning to project multiple radiotherapy beams and create a highly conformal plan to deliver high dose to the tumour. The number of beams and their orientation varies depending on local centre techniques and tumour location. Higher doses are expected along the path of the beams, and these can be chosen to avoid organs at risk, although this is not always possible. Volumetric modulated arc therapy (VMAT) techniques are also used for lung radiotherapy, typically leading to greater target conformality but with larger volumes exposed to low/intermediate dose levels. Mandatory dose constraints such as the Dmax to spinal cord and lung V20 Gy are employed to reduce the risk of known toxicity such as myelopathy and pneumonitis, respectively. This results in dose being delivered to nearby organs such as the heart, which is often not prioritised in the planning constraints. A dosimetry study evaluated CS sparing treatment plans in central and ultra‐central lung tumours and found it was possible to significantly reduce the dose to the LA and D4% RV, without compromising target coverage or increasing other organ at risk (OAR) tolerances. Plans were more likely to require cardiac sparing if the tumour was in the same axial plane as the LA.^59^

### Oesophagus

IMRT and VMAT techniques are also used in oesophageal cancer. Evidence has shown that cardiac death was significantly more likely in patients who received radiotherapy to the lower oesophagus, compared with the upper and middle third of the oesophagus.^60^ IMRT and VMAT can reduce heart dose, but this may be achieved at the expense of increased mean lung dose, which has been shown to increase risk of pneumonitis, with an adverse impact on OS.^61^ There are fewer modelling and data mining study results available in oesophageal cancer. The results from breast and lung cancer studies cannot be extrapolated to this patient group due to the different anatomical position of the high dose region.

## How can the dose to cardiac substructures be reduced?

Current radiotherapy techniques can be utilised to achieve reduction in dose to CS. One study used VMAT to re‐optimise the plans of 20 patients with NSCLC to limit the dose to the LA, based on the IDEAL‐CRT suggested LA dose limit of 63 Gy.^62^ This was easily achieved whilst maintaining target coverage and OAR constraints. They showed a reduction in LA V63 Gy, by 2.4% absolute or 96% of mean baseline value, with insignificant increase in MLD and decrease in tumour coverage overall. 90% coverage of the planning target volume (PTV) with ≥ 95% of the prescribed dose at baseline was 98.6% compared with 98.4% after LA dose reduction technique (*P* = 0.31). Similarly, the dose to CS such as LAD V15 Gy was significantly reduced using a knowledge‐based planning system that was trained using plans optimised to reduce CS dose. Plans generated using this system had reduced CS doses, whilst maintaining tumour coverage and OAR doses. LAD V15 Gy was 0.69 cc (±1.57 cc) versus 1.23 cc (±1.76 cc) compared with manually/clinically optimised plans and LV V30 Gy was also reduced (1.54 cc vs 9.44 cc).^63^

**Fig. 2:**
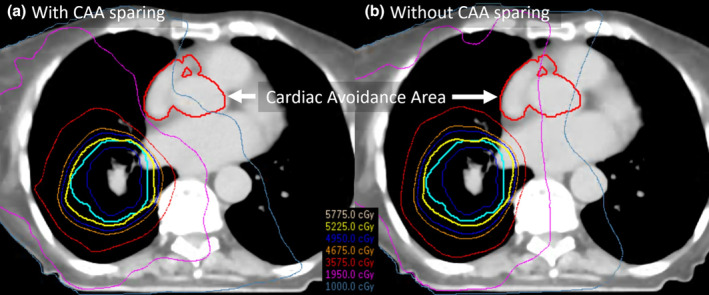
Comparison of lung RT plans optimised (a) with and (b) without objectives to reduce maximum dose to base of heart cardiac avoidance region.

Due to the unclear evidence on the dose parameters for specific cardiac substructures, avoiding a composite structure may be beneficial for patient outcomes. Figure 2 presents a comparative lung plan optimised (a) with and (b) without dose‐sparing objectives for a composite cardiac avoidance area (CAA) located in the base of heart. This incorporates the right atrium, aortic valve root and proximal left and right coronary arteries. These plans were generated using a dual arc VMAT technique, which facilitates the sculpting of dose distribution to minimise CAA exposure. The planning objective was to limit CAA maximum dose to less than 19.5 Gy. The isodoses shown in Figure 2a illustrate that this is achieved, while in Figure 2b much of the CAA receives doses between 19.5 and 35 Gy.

Integrating additional avoidance structures can increase multileaf collimator modulation and monitor units. The increased plan complexity may lead to hotspots within the dose distribution or compromise the robustness of plan deliverability.^64^ Consequently, rigorous plan quality assurance (QA) is important to validate the acceptability of the final plans. Proximity of the target volume to the CAA will also determine the degree of dose reduction possible. New strategies are required to deal with overlap or adjacency of target volume with CS. For patients with middle third oesophageal cancer, where the PTV is in close proximity to the heart, DIBH significantly reduced the dose to several CS compared with free breathing.^65^

Furthermore, dose reduction in one area may inadvertently elevate it in another. For instance, the plans depicted in Figure 2 exhibit a marginal dose increment to the spinal cord following the implementation of CAA dose‐sparing. Additionally, the inherent motion of heart substructures due to respiratory or cardiac cycles introduces positional and therefore dose uncertainty.^66^ Hence, the adoption of Planning Organ at Risk Volumes (PRVs) is recommended, especially for small or narrow structures like the coronary arteries, to accommodate such variability.

Proton beam therapy (PBT) may be of benefit in thoracic radiotherapy, as the rapid dose fall off reduces dose to nearby OAR and low dose scatter. This can allow escalation of dose while limiting the dose to CS.^67^ PBT has shown reduced dose to CS in oesophageal, breast and lung cancers.^68^, ^69^, ^70^, ^71^ The ventricular and LAD dose were significantly lower in proton beam therapy versus photon therapy in NSCLC. However, doses to other substructures such as RA and SVC were not significantly different and lower substructure doses did not translate into lower rate of cardiac events in the group who received PBT.^71^ This was a small retrospective analysis of patients who experienced cardiac events following proton or photon therapy (*n* = 26), which may have limited the analysis. Another trial in patients with NSCLC found that intensity‐modulated proton therapy (IMPT) (*n* = 35) was associated with fewer number of ≥G3 cardiac events at 1 year (11.6% vs 0%). Although potentially clinically significant, this was not statistically significant, and it did not translate into OS benefit.^72^

A meta‐analysis of use of proton therapy in oesophageal cancer, showed a significantly reduced dose to CS particularly the LV and LAD.^73^ Dose to the LV was reduced by 10.2 Gy and LAD by 11.2 Gy. OS was higher in those treated with protons compared to photons (HR 1.31, 95% CI 1.07–1.61, *I* ^2^ = 11%). Varying proton techniques are available, with IMPT (vs passive scatter techniques) previously shown to reduce >G3 cardiopulmonary toxicity.^74^ Several phase three RCTs are currently underway to explore this topic further with cardiac‐specific endpoints; RTOG‐1308 (NSCLC),^75^ NRG‐G1006 (Oesophagus)^76^ and RADCOMP (Breast)^77^ and PARABLE (Breast).^78^

The use of MR‐guided radiotherapy to spare CS is also being evaluated. The advantage of MR‐based therapy is accurate daily visualisation of cardiac anatomy, allowing greater precision. Very small set up shifts have been shown to increase mortality in patients with lung cancer (HR for death 1.26 per 1 mm shift towards the heart).^79^ MR‐based imaging could improve CS visualisation, compared with CT‐based imaging at the time of radiotherapy planning. One study applied multimodality imaging including images taken on an MR‐linear accelerator into CT‐based treatment planning for pericardial tumours (lung, mediastinum and oesophagus) to assist in plan re‐optimisation for CS sparing. It was possible to reduce the cardiac dose to 16 patients' plans. The MHD and dose to all CS were significantly reduced. The LAD mean dose was reduced by up to 4 Gy, and max dose to LAD reduced by up to 17.3 Gy, dose to the LV was reduced by up to 12.9 Gy.^80^

The MHD remains consistent throughout the cardiac cycle unlike the dose to the LAD, which varies throughout the cardiac cycle and is lowest in systole.^81^ Cardiac gating is a technique used to evaluate the position of CS during end‐diastolic and end‐systolic phases of the cardiac cycle via electrocardiogram monitoring. A study of cardiac motion effect found most significant difference in the ventricle mean doses. There were morphological and volumetric differences in the CS during the cardiac cycle. This again demonstrates the need for evaluation of a PRV to account for the uncertainty in position of CS.^82^

Despite the advantages of cardiac sparing radiotherapy, it is difficult to adequately power a randomised controlled trial to demonstrate the causal effect of reduced cardiac radiation and improved OS as, due to the small differences in outcome, large numbers of patients would be required with a long duration of follow‐up.^62^ Reduced dose to the base of the heart has been introduced for all patients receiving curative, non‐SABR radiotherapy at one UK centre and the outcomes of this including cardiac toxicity and survival will be recorded prospectively allowing contemporaneous assessment of real‐world outcomes.^83^

## Discussion

There is emerging evidence to suggest that the use of MHD may no longer be the most appropriate dose parameter for thoracic radiotherapy and that dose to CS is increasingly relevant to our understanding of RICD. Nevertheless, there is no consensus in the literature regarding which CS is of optimal importance or universally agreed dose constraints. Current evidence is at times conflicting due to heterogeneity of study populations included, the retrospective nature of studies and the lack of universally agreed cardiac endpoints. At the time of writing there were 214 cardiac toxicity endpoints being used in research.^84^

Contouring of cardiac substructure can also vary between studies. IBDM provides contour‐free, hypothesis generating data on dose sensitive CS. Areas demonstrated on IBDM have been successfully taken from pre‐clinical studies to a prospective clinical study in patients with lung cancer.^83^ To the best of our knowledge, no IBDM analysis has been performed in patients with breast or oesophageal cancer to determine which areas are most sensitive to the effects of radiation. As IBDM methodology is expanded, it may be useful to apply to this group of patients.

## Conclusion

Emerging evidence highlights the need to evaluate and reduce the dose delivered to cardiac substructures, moving beyond the traditional MHD model. Despite this, a consensus on the most appropriate dose constraints for CS, tailored to the disease site being treated and patient factors has yet to be reached. Consequently, CS avoidance should currently only be performed as part of clinical trials. In the absence of clear evidence on the balance of risks between tumour dose and CS sparing, clinicians and radiotherapy planners should continue to prioritise target coverage. Further research is necessary to establish evidence‐based, universally accepted dose constraints. Future trials should integrate dosimetry information, cardiac imaging, advanced radiotherapy techniques, patient comorbidities and high‐quality toxicity data. Creating and validating these models could lead to personalised radiotherapy techniques, based on individualised cardiac risk profiles, to be employed for patients to mitigate the risk of radiation‐related cardiotoxicity.

## Funding

This work was supported by the Cancer Research UK Manchester Centre award [CTRQQR‐2021\100010] and Cancer Research UK RadNet Manchester [C1994/A28701]. This project was also funded by the National Institute for Health Research (NIHR) under its Programme Grants for Applied Research Programme (NIHR202024). The views expressed are those of the author(s) and not necessarily those of the NIHR or the Department of Health and Social Care. Alan McWilliam is supported by NIHR Manchester Biomedical centre.

## Data Availability

Data sharing is not applicable to this article as no new data were created or analyzed in this study.
